# Non-Destructive, Opto-Electronic Determination of the Freshness and Shrivel of Bell Pepper Fruits

**DOI:** 10.3390/jimaging6110122

**Published:** 2020-11-10

**Authors:** Bernhard Althaus, Michael Blanke

**Affiliations:** INRES-Horticultural Science, Faculty of Agriculture, University of Bonn, D-53121 Bonn, Germany; bernhard.50668@web.de

**Keywords:** bell pepper (*Capsicum annuum* L.), agriculture 4.0, color, digitalization, freshness, false color image, fruit quality, gloss, light reflection, roughness, shelf-life, storage, *Solanaceae*

## Abstract

(1) The objective of the present study was to identify suitable parameters to determine the (degree of) freshness of Bell pepper fruit of three colors (yellow, red, and green) over a two-week period including the occurrence of shrivel using non-destructive real-time measurements (2) Materials and methods: Surface glossiness was measured non-destructively with a luster sensor type CZ-H72 (Keyence Co., Osaka, Japan), a colorimeter, a spectrometer and a profilometer type VR-5200 (Keyence) to obtain RGB images. (3) Results: During storage and shelf life, bell pepper fruit of initially 230–245 g lost 2.9–4.8 g FW per day at 17 °C and 55% rh. Shriveling started at 6–8% weight loss after 4–5 days and became more pronounced. Glossiness decreased from 450–500 a.u. with fresh fruit without shrivel, 280–310 a.u. with moderately shriveled fruit to 80–90 a.u. with severely shriveled fruit irrespective of color against a background of <40 a.u. within the same color, e.g., light red and dark red. Non-invasive color measurements showed no decline in Lab values (chlorophyll content), irrespective of fruit color and degree of shrivel. RGB images, converted into false color images, showed a concomitant increase in surface roughness (Sa) from Sa = ca. 2 µm for fresh and glossy, Sa = ca. 7 µm for moderately shriveled to Sa = ca. 24 µm for severely shriveled rough surfaces of stored pepper fruit, equivalent to a 12-fold increase in surface roughness. The light reflectance peak at 630–633 nm was universal, irrespective of fruit color and freshness. Hence, a freshness index based on (a) luster values ≥ 450 a.u., (b) Sa ≤ 2 µm and (c) the difference in relative reflectance in % between 630 nm and 500 nm is suggested. The latter values declined from ca. 40% for fresh red Bell pepper, ca. 32% after 6 days when shriveling had started, to ca. 21% after 12 days, but varied with fruit color. (4) Conclusion: overall, it can be concluded that color measurements were unsuitable to determine the freshness of Bell pepper fruit, whereas profilometer, luster sensor, and light reflectance spectra were suitable candidates as a novel opto-electronic approach for defining and parametrizing fruit freshness.

## 1. Introduction

Bell pepper fruit have a short storage and shelf-life among vegetables, are cold temperature sensitive and as a non-climacteric fruit insensitive to ethylene (Kader, 1999 [1]; Blanke and Holthe, 1997 [2]). Pepper fruit are special, because their peel lacks stomata [2,3] in contrast to the majority of fruits (Blanke and Lenz, 1989) [4]. Hence, water loss through the cuticle is the exclusive source of water and weight loss and shriveling. Parameters for the state of freshness have long been discussed for pepper fruit (Kays, 1999; Salunkhe and Desai, 1984) [5,6]. In the apparent absence of studies evaluating non-destructive measurements to determine fruit freshness in Bell pepper, the objective of the present study was to identify suitable parameters to determine the (degree of) freshness of bell pepper fruit over a two-week period making use of a new profilometer and a new gloss sensor (Czieczor et al., 2017) [7], which enable repeated real-time in situ measurements on the same spot on the same fruit.

## 2. Materials and Methods

### 2.1. Fruit Source

**Figure 1:**
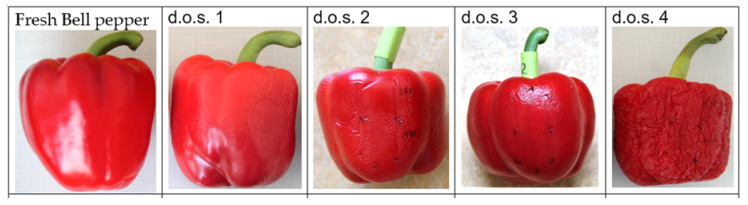
Our designation of shriveling of the bell pepper fruit deployed for the experiment defined as degree of shriveling (d.o.s.).

**Figure 2:**
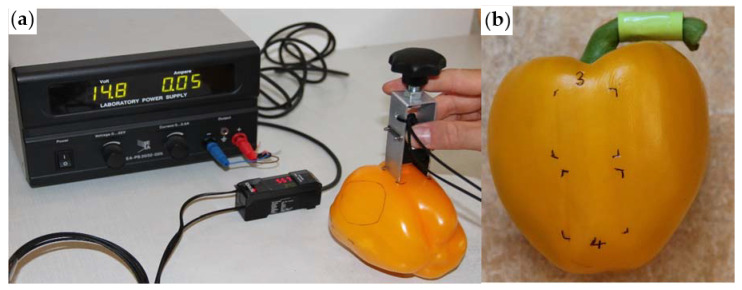
(**a**) Gloss measurements using our designed machined holder (left) and (**b**) marked spots/areas for repeated non-invasive measurements at the same positions (right).

Freshly delivered Bell pepper fruits of Spanish origin without surface defects such as bruises were obtained in May 2020—during Covid-19—from a local supermarket. Sixty bell pepper fruits were purchased, i.e. 20 fruit for each color allowing 19 replicates for each measurement. Fruit were stored at ca 17 °C and 55% rh over two weeks and carefully examined for their shriveling degree (Figure 1). The same spots on the center of the convex side of the fruit were marked for repeated non-invasive measurements of gloss, roughness (by false color images), color and spectra (Figure 2).

### 2.2. Gloss Measurements 

Surface glossiness of the Bell pepper fruit was determined by a non-invasive technology using a CZ-H72 luster sensor (Keyence, Co., Osaka, Japan). The sensor provides red LED light at 665 nm. The operating voltage was 14.8 V. The measuring spot was 5 mm diameter. In contrast to previous work, which required a micromanipulator (Klemm et al., 2016) [8], a holder (Figure 2a) was design-ed to provide a constant distance of 15 mm between the luster sensor and the fruit without the necessity of a micromanipulator and without moving the fruit and provides the luster values in real time.

### 2.3. Color Measurements

The peel color of the Bell pepper fruit was measured with a non-invasive colorimeter (380–720 nm) using i1Pro (X-Rite, Grand Rapids, MI, USA) with a resolution of 10 nm and a measuring spot size of 3.5 mm diameter. Color values L (brightness), a (green to red) and b (yellow) in die CIE-Lab color space were converted to the color angle °hue according to McGuire (1992) [9].

### 2.4. Spectrometry

**Figure 3:**
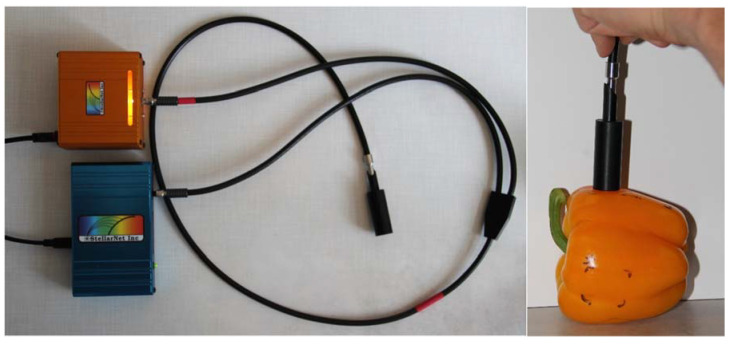
Light reflectance measurements (0° from the normal) with a portable spectrometer.

Spectral reflection of the pepper fruit with different degrees of freshness and shrivel were measured non-invasively with a portable miniature spectrometer (StellarNet Inc., Tampa, FL, USA) with data processing using SpectraWiz^TM^ supplied with the instrument. Measurements over 500–850 nm using the build-in halogen light source at 0° (from the normal) reflection angle (Figure 3) with a resolution of 0.2–6.0 nm depending on wavelength and a flexible glass fiber cable to provide a measuring spot of 2 mm with a measuring time of less than 1 s. The instrument was calibrated with the supplied 5 cm O white barium sulfate disc as reference for 100% light reflection and the data expressed as percent in Figure 5.

### 2.5. Profilometer for Roughness Determination Using 3D False Color Images

In high resolution (2 µm) digital microscopy, the 3D profilometer VR-5200 (Keyence, Osaka, Japan) is the latest technology for non-invasive examination of, by microscopic standards, relatively large surfaces (200 mm × 100 mm). Previous instruments measured roughness, e.g., of pomegranate fruit (Czieczor et al., 2017) [7] of individual lines but not squares. The VR-5200 provides the high resolution RGB pictures, which are converted into false images of the surface of bell pepper fruit. These false color images are then used to determine the physical roughness parameters Sa (arithmetic difference between peaks and troughs) and Sz (maximal heights) (Keyence, Osaka, Japan 2020) as

(1) $$Sa=\frac{1}{A}\iint_{A}^{k}\left|Z\left(x,y\right)\right|dxdy$$

(2) Sz=Sp+SvSp=max(Z(x))Sv=|min(Z(x))|

where Sp is the maximum peak height, Sv is the maximum trough height, Sz is the maximum height and Z is the ‘ten-peak-height’.

### 2.6. Statistics

**Figure 4:**
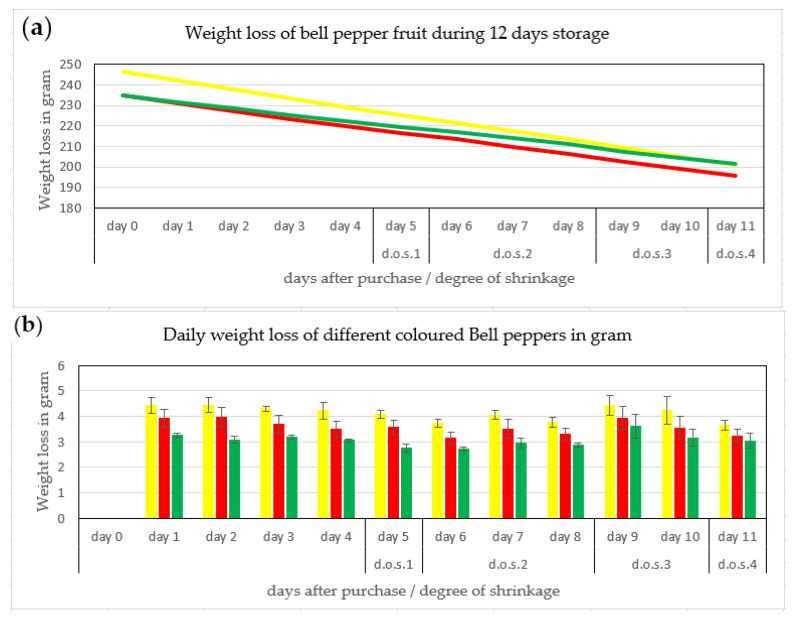
(**a**) Weight loss of bell pepper fruit during 12 days storage (top) and (**b**) daily weight loss (bottom) (averages of yellow, red and green fruits ± SE) and the degree of shriveling. (d.o.s. 1 begin of shriveling, d.o.s 2 slight shriveling, d.o.s 3 moderate shriveling, d.o.s 4 severe shriveling/ discarded for human fresh fruit consumption) (n = 60 pepper fruit).

**Figure 5:**
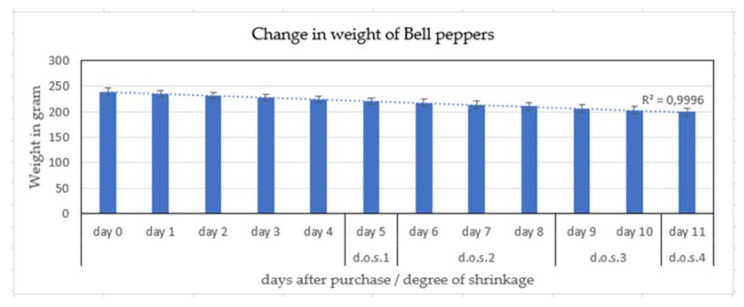
Weight loss of bell pepper fruit stored for 14 days averaged over all three fruit colors and considering the degree of shrinkage (d.o.s.) (n = 60 pepper fruit).

**Figure 6:**
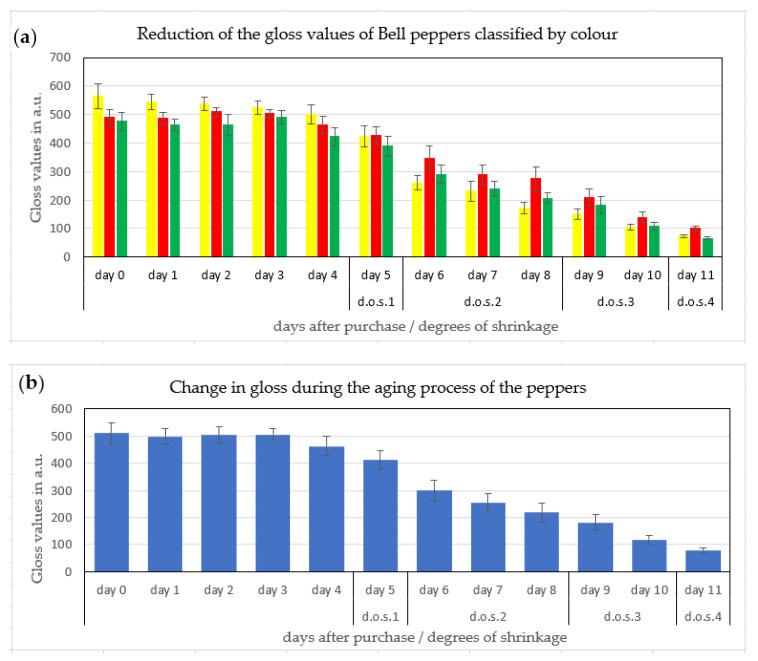
(**a**) Loss of the gloss of bell peppers classified by color (top) (n = 20 per color) and (**b**) averaged gloss values per day, stored over 14 days (bottom) (n = 60 pepper fruit per day; ± SE).

After testing the experimental data for normal distribution (Kolmogorov-Smirnov test and Shapiro-Wilk test) and for variance homogeneity (Levene test), they were statistically processed using one-factorial analysis of variance (ANOVA), using aging as group/factor within one fruit color (Figure 4 and Figure 6a) or within all 60 Bell pepper fruit (Figure 5 and Figure 6b) in RStudio (Boston, MA, USA, version 1.3) at 95% probability.

## 3. Results

### 3.1. Weight Loss of Pepper Fruit

Fresh bell pepper fruit with 222–280 g weight (Figure 4a) showed a linear weight loss of 3.1–4.8 g per fruit per day (Figure 4) for the two weeks of the experiment, which is equivalent to ca. 2% weight loss per day. This rate of this predominantly moisture loss through the cuticle [2] and possibly cuticular cracks [3] remained constant during 14 days of storage. 

Visual observation showed that fruit shriveling began on day 5 (Figure 4), i.e., after 6–8% weight loss. If the individual values in Figure 4b are averaged, the trend line showed that the daily weight loss was linear (Figure 5). 

### 3.2. Gloss Disappearance of the Bell Pepper Fruit—A Possible Freshness Indicator? 

Gloss and turgidity represent the attractiveness of the fruit as perceived by the human eye [4]. During two weeks of storage, the gloss of bell pepper fruit deteriorated (Figure 1). Non-invasive, real time gloss measurements in situ using a luster sensor type CZ-H72 (Keyence, Osaka, Japan) enabled differentiation between different stages of gloss in accordance with the degree of shrivel (Figure 6a). 

### 3.3. Color Change of the Bell Pepper Fruit

**Figure 7:**
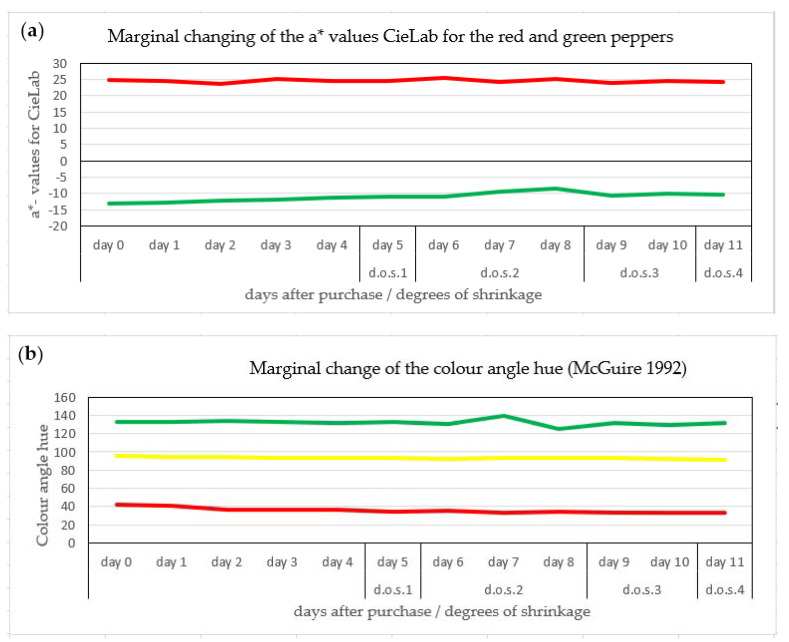
(**a**) Lack of measurable color change in harvested bell pepper fruit, expressed as a* color values (red or green) of red and green peppers and (**b**) hue color angle of red, green, and yellow peppers during 14 days of storage (n = 2 measurements per fruit per day).

Non-invasive color measurements showed a negligible increase in a* values for green bell pepper fruit from −15 to −10 (Figure 7a), indicating the marginal loss of chlorophyll during storage. Similarly, there was no marked change in color for the red and yellow Bell pepper fruit (Figure 7b). The apparent lack of color change in Bell pepper fruit in storage excludes the color measurements as a suitable indicator for the fruit freshness, irrespective of their color. 

### 3.4. Increase in Surface Peel Roughness—Derived from 3D False Color Images of the Profilometer

**Figure 8:**
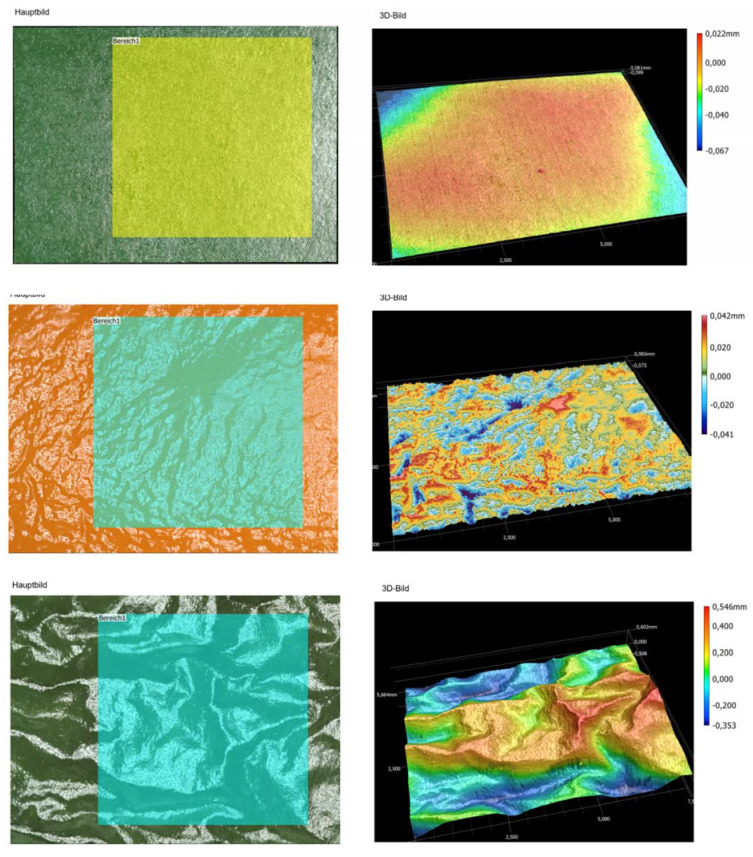
RGB (left column) and false color images (right column) of fresh, glossy (top), begin of shrivel after day 6 (d.o.s.2) (middle) and severely shriveled (bottom) pepper fruit indicating the degree of roughness between red (peaks) and blue (troughs) in these false color images; magnification × 40.

The concomitant results of decreasing gloss (Figure 6) and beginning of shrivel with storage of the pepper fruit (Figure 1) can be explained by increasing peel roughness. The roughness values increased from Sa = ca. 2 µm (fresh, glossy fruit), Sa of ca. 7 µm for slightly shriveled fruit to Sa = 27.9 µm (severely shriveled fruit surface) (Table 1/Figure 8); expressed in a more than 12-fold increase in surface roughness (Table 1) over the 2 weeks of storage and increase in blue color (Figure 8).

### 3.5. Spectral Light Reflectance—Development of a Freshness Index 

**Figure 9:**
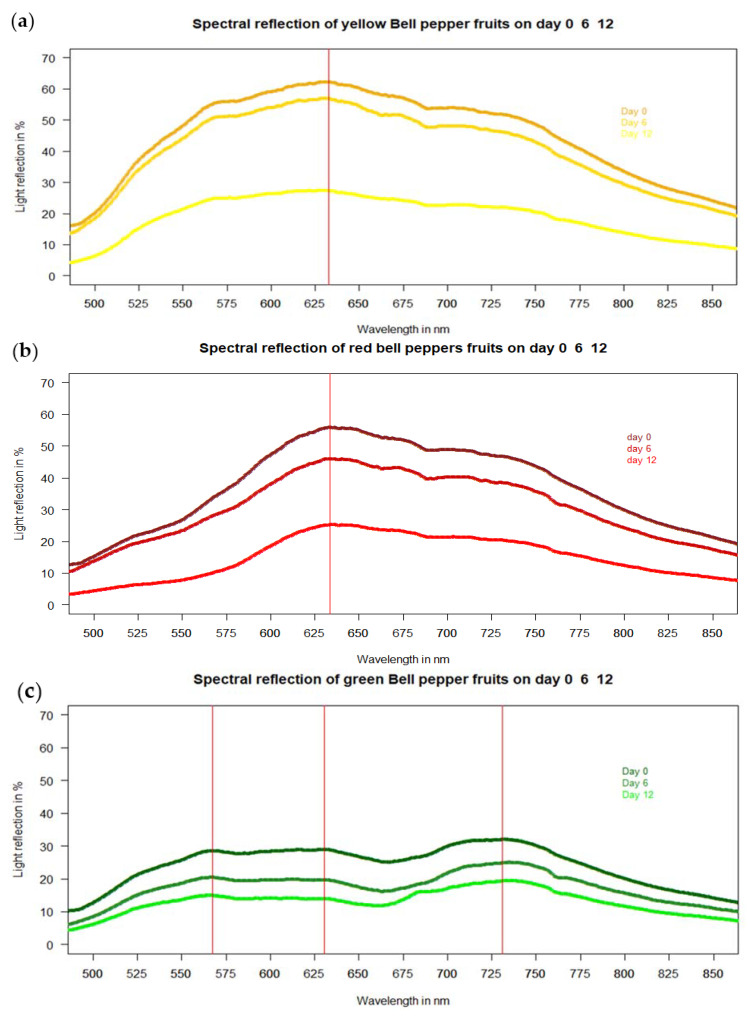
Percentage spectral light reflection of (**a**) yellow, (**b**) red, and (**c**) green bell pepper fruits in three stages of freshness (shriveling and storage day 0 = fresh fruit, day 6 = d.o.s 2, day 12 = d.o.s 4) (barium sulfate = 100% light reflectance) (n = 20 fruit per color, i.e., per curve; n = 60 overall).

Light reflection was measured on the convex side of the fruit in situ, non-destructively and in real time with a portable unit. The magnitude of reflected light between 500 and 850 nm declined depending on storage of bell pepper fruits, but irrespective of fruit color (Figure 9). Yellow bell pepper fruits showed the largest light reflection (Figure 9a), followed by red (Figure 9b), and then green fruit (Figure 9c). The spectra showed one uniform reflectance peak at the same wavelength of 630–632 nm, irrespective of fruit color and freshness. However, the degree of light reflection declined with freshness in line with the glossiness values (Figure 6), irrespective of fruit color. Hence, a new freshness index is suggested in combination with luster values < 450 a.u., Sa > 2 µm and the difference of the light reflectance between 630 nm and 500 nm using the following formula:

(3) Freshness index=Reflectance 630 nm−Reflectance 500 nm

Freshness may be parametrized by the universal reflectance peak at 630–633 nm and reflectance at 500 nm (formula (3)). With fresh red pepper fruit, the difference in reflectance between 630 nm and 500 nm is 40.4%, declined to 32.0% with 6-day old peppers (shriveling degree-d.o.s. 2) and finally to ca. 20.7% with 12-day old fruits (shriveling degree-d.o.s. 4) (Table 2).

**Figure 10:**
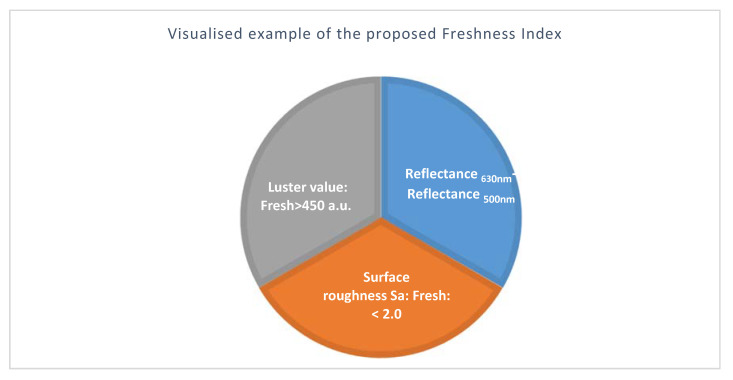
Visualized example for a possible freshness index, integrating the current findings (Figure 6, Figure 9, and Table 2) and two or three parameters depending on available instrumentation and situation (transport, storage, retail); spectral values (Table 2)/thresholds depend on fruit color.

Figure 10 presents an example for a freshness (or shrivel) index for bell pepper fruit.

## 4. Discussion

For retail, the fresh fruit market and the consumer, determination of freshness in often requested [1]. Apart from size and color, gloss, turgidity and a smooth surface determine the attractiveness of the fruit as perceived by the human eye [5]. In our experiment, shriveling started at 6.5% (green)—8.7% (yellow) weight loss (Figure 1 and Figure 4) in line with results of 6% also with Bell pepper of Hiepler (2004) [10] stored under a colder temperature (3.4 °C; 90% rh) in her experiments compared to our investigation (17 °C; 55% rh).

The hollow volume of Bell pepper fruit, designated botanically as ‘interlocular space’ by the German-Californian authors [2], was examined for its gaseous composition and contained ca. 19.0% O_2_ (Oomens et al., 1998) [11] confirming the original values of 19.2(±0.1)% oxygen and 1.8% CO_2_ in MexiBell pepper fruit at harvest in the pioneering work of Blanke and Holthe (1997) [2]. This CO_2_ concentration resembles that in efficient long-term CA storage, but hampered by the high oxygen concentration.

Our results have shown (Figure 4) that the conspicuous Bell pepper fruit color changed marginally, e.g. the dominant fruit color (yellow, red, green) remained, and in red and yellow pepper fruit as a result of chloroplast to chromoplast transition (Ziegler et al., 1983) [12]. The apparent maintenance of the chlorophyll content is in contrast to dramatic chlorophyll degradation in many other fruits such as apple [3], cherry (Overbeck et al., 2017) [13], or banana (Ringer et al., 2018) [14].

Freshness has long been discussed, but time has come now to utilize current opto-electronic approaches. In the past, freshness was sometimes judged by the visual appearance (moist or dry) of the stem cut, e.g., in pepper, asparagus, green celery, cabbage etc.; similar new approaches have recently been applied to russet [15] and also included the development of a russet index derived from spectral light reflection and changes in its light peaks and troughs.

Pepper belongs to the *Solanaceae* family. Their fruits—such as tomato, pepper, and aubergine—are a dominant vitamin and mineral source in human nutrition worldwide and characterized by glossy fruit at maturity and absence from stomata from these three fruits (Blanke, 1986) [16]. For the related aubergine (eggplant), a gloss index (GI) was developed by Mizrach et al. (2009) [17] for fresh apple, nectarine, plum, and tomato fruit, which is comparable to our approach with fresh and stored Bell pepper. Mizrach et al. (2009) [17] used a laboratory set-up in Michigan (USA) and integrated the area underneath the gloss curves from 500 nm to 780 nm for both high gloss (probably waxed) US American Red Delicious and low gloss green-yellow Golden Delicious apples similar to our VIS light reflection spectra for Bell pepper fruit in Figure 9. The spectra of Mizrach et al. (2009) [17] showed a similar light reflection peak at 630–640 nm using fresh Delicious apple fruit without storage or different degree of freshness and shrivel. Fruit with a high degree of glossiness such as the waxed US American Red Delicious also showed a higher light reflectance than low gloss yellow-green Golden Delicious in line with our findings 10 years later with fresh and stored Bell pepper fruit, which lost glossiness over time (Figure 6, Figure 7, Figure 8 and Figure 9) taking Mizrach’s original ideas at the time into the next century.

## 5. Conclusions

Overall, this is the first approach to employ real-time, non-destructive in situ measurements for the determination of freshness of certain fruits. This novel opto-electronic approach is based on surface features of certain fruits such as peel gloss, surface roughness, and shrivel. Bell pepper was used as a model fruit. The results including a freshness index may be transferable to other fruit, which exhibit these features.

## Figures and Tables

**Table 1:** Roughness values Sa and Sz (in µm) of green pepper fruit during 2 weeks storage

Degree of Freshness and Shrivel	Sa (µm)	Sz (µm)
Fresh, glossy pepper—d.o.s. 0	1.9	82
Slight shrivel on day 6— d.o.s 2	7.1	93
Severe shrivel on day 12—d.o.s. 4	23.9	373

**Table 2:** Freshness index (=difference between light reflection at 630 nm and 500 nm)

Colour of Bell Pepper	Day 0 (Fresh)	Day 6 (d.o.s. 2)	Day 12 (d.o.s. 4)
Yellow Bell pepper	42,2%	38.6%	21.1%
Red Bell pepper	40.4%	32.0%	20.7%
Green Bell pepper	16.2%	11.1%	7.8%
